# Measuring the effects of motion corruption in fetal fMRI

**DOI:** 10.1002/hbm.26806

**Published:** 2025-01-23

**Authors:** Athena Taymourtash, Ernst Schwartz, Karl‐Heinz Nenning, Roxane Licandro, Patric Kienast, Veronika Hielle, Daniela Prayer, Gregor Kasprian, Georg Langs

**Affiliations:** ^1^ Computational Imaging Research Lab, Department of Biomedical Imaging and Image‐Guided Therapy Medical University of Vienna Vienna Austria; ^2^ Center for Biomedical Imaging and Neuromodulation, Nathan Kline Institute Orangeburg New York USA; ^3^ Laboratory for Computational Neuroimaging, A.A. Martinos Center for Biomedical Imaging, Massachusetts General Hospital and Harvard Medical School Charlestown Massachusetts USA; ^4^ Division of Neuroradiology and Muskuloskeletal Radiology, Department of Biomedical Imaging and Image‐Guided Therapy Medical University of Vienna Vienna Austria; ^5^ Computer Science and Artificial Intelligence Laboratory Massachusetts Institute of Technology Cambridge Massachusetts USA

**Keywords:** artifact, fetal fMRI, functional connectivity, in‐utero, motion, noise, resting‐state

## Abstract

Irregular and unpredictable fetal movement is the most common cause of artifacts in in utero functional magnetic resonance imaging (fMRI), affecting analysis and limiting our understanding of early functional brain development. The accurate detection of corrupted functional connectivity (FC) resulting from motion artifacts or preprocessing, instead of neural activity, is a prerequisite for reliable and valid analysis of FC and early brain development. Approaches to address this problem in adult data are of limited utility in fetal fMRI. In this study, we evaluate a novel technique for robust computational assessment of motion artifacts, and the quantitative comparison of regression models for artifact removal in fetal FC analysis. It exploits the association between dynamic FC and non‐stationarity of fetal movement, to detect residual noise. To validate our motion artifact detection technique in detail, we used a parametric generative model for neural events and fMRI blood oxygenation level‐dependent (BOLD) signal. We conducted a systematic evaluation of 11 commonly used regression models in a sample of 70 fetuses with gestational age of 19–39 weeks. Results demonstrate that the proposed method has better accuracy in identifying corrupted FC compared to methods designed for adults. The technique, suggests that censoring, global signal regression and anatomical component‐based regression models are the most effective models for compensating motion. The benchmarking technique, and the generative model for realistic fetal fMRI BOLD enables investigators conducting in utero fMRI analysis to effectively quantify the impact of fetal motion and evaluate alternative regression strategies for mitigating this impact. The code is publicly available at: https://github.com/cirmuw/fetalfMRIproc.

## Graphical abstract

Illustration of the systematic approach to measure the relationship between functional connectivity (FC) and motion at the subject level. The average time series of blood oxygenation level‐dependent signals from cortical ROIs are extracted. Time‐varying functional connectivity and framewise displacement (FD) are computed over sliding windows and the correlation coefficient is measured between them (a). To evaluate the statistical significance of the time‐varying FC–FD relation, an appropriate null distribution is formed by generating surrogate FD time series and repeating the entire procedure (b). Comparing the true value of the time‐varying FC–FD relation to the null distribution determines the extent to which motion drives the changes in FC.

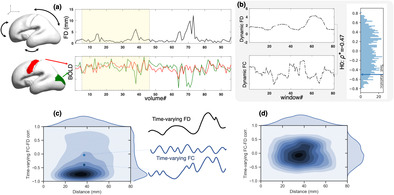

## 1 INTRODUCTION

Functional magnetic resonance imaging (fMRI) has emerged as a powerful tool to evaluate the functional organization of the brain and its variation due to development, aging, and neuropsychological disorders. While fMRI identifies consistent patterns of functional connectivity (FC) across individuals (Damoiseaux et al., 2006; Smith et al., 2009), it is highly sensitive to artifacts and physiological noise arising from heart rate, respiration, or head motion (Krüger & Glover, 2001). Even submillimeter movements can induce spurious variance to blood oxygenation level dependent (BOLD) signal (Glover et al., 2000), and systematically bias between‐group differences (Power et al., 2012; Satterthwaite et al., 2013; Yan et al., 2013). This problem is particularly pronounced in the fetal population and in utero imaging since fetal movements are unconstrained and potentially substantial. Additionally, heterogeneous maternal tissues and placenta surrounding the fetal head generate true physiological signals resulting in a much lower signal‐to‐noise ratio in fetal BOLD signals (Vasung et al., 2019). Therefore, artifact removal techniques developed for adult fMRI do not translate directly to fetal scans, and are a topic of research.

As methods to counteract motion artifacts are being developed (Liao et al., 2019; Rutherford et al., 2022; Scheinost et al., 2018; Seshamani et al., 2016; Sobotka et al., 2022; Taymourtash et al., 2022; You et al., 2016), it is of critical importance to know if a technique has improved the quality of data or introduced additional artifacts. In response to the proliferation of preprocessing approaches, several studies have compared the effectiveness of different pipelines in the adult population (Ciric et al., 2017; Fair et al., 2020; Kassinopoulos & Mitsis, 2022; Lydon‐Staley et al., 2019; Parkes et al., 2018) and children (Graff et al., 2022), but no study has specifically considered the fetal population and in utero imaging data. The widely used benchmarking metric of quality control–FC (QC–FC) that is specific to head motion (Power et al., 2014) is of only limited applicability in fetal data for several reasons. First, relying on the average observed motion throughout the entire scan, as employed in this metric, might not reliably and adequately capture the continuous and abrupt movements often exhibited by fetuses. Second, the smaller head size of fetuses highlights the effects of rotational motion, and biases FC toward stronger short‐range connections (Jakab et al., 2014; Van den Heuvel & Thomason, 2016). Since many artifact removal approaches showed distance‐dependent effects, the result of preprocessing approaches may interact with the brain size that significantly changes during gestation. Furthermore, the characteristics of physiological noises differ significantly between fetuses and adults. The average fetal heart rate is between 110 and 160 beats per minute (bpm) and breathing movement rates range from 30 to 70 bpm. As a result, their contribution to the cross‐correlation coefficient and FC maps will be different for this specific population.

An alternative metric that offers a subject‐specific assessment of signal retention is intra‐class correlation (ICC, Shrout & Fleiss, 1979) which evaluates test–retest reliability in longitudinally acquired data. A denoising strategy that successfully maintains a stable and reproducible estimation of FC over repeated measurements of the same subject under the same conditions would be expected to yield higher ICC values (Parkes et al., 2018). However, given the maturational processes and rapid conformational changes of the developing brain even within several days (Bystron et al., 2008), the ICC metric is likely to be heavily influenced by the substantial variability in fetal data. A recent work investigating functional fingerprints of preterm born neonates who were scanned soon after birth and then again at term equivalent age demonstrated that only 10% of the participants had greater self‐similarity in comparison to self‐to‐other‐similarity in their functional connectome, suggesting FC is being too dynamic or immature in early development to provide a reliable fingerprint (Ciarrusta et al., 2022). Another study showed low uniqueness and stability of FC patterns (i.e., fingerprint) with the poorest ICCs across infancy which may reflect the rapid and unparalleled development of the brain during this time (Dufford et al., 2021). Moreover, previous work on benchmarking denoising strategies in adult fMRI (Kassinopoulos & Mitsis, 2022; Parkes et al., 2018) have counterintuitively found that higher ICC is obtained in less effective methods of denoising, suggesting a substantial fraction of reproducible BOLD signal is driven by head motion and/or other physiological confounds.

Subject‐level QC metrics allow researchers to make informed decisions about whether to include or exclude a particular individual from the analysis. This is particularly important in fetal fMRI studies where data quality can vary substantially due to individualized factors such as fetal motion, maternal motion, and variations in brain size. Given the lack of consensus on the most effective methods for mitigating motion artifacts, or even on the means of assessing effectiveness among different approaches in fetal population, we sought to develop a computational QC method to automatically quantify the residual effects of motion on FC at the subject level. We demonstrated that traditional assessment methods may not adequately capture the relationship between fetal motion and FC, and can potentially create a misleading reassurance of data quality despite the presence of motion artifacts. Since there is no ground truth for the brain's functional structure, especially before birth, in addition to evaluation on fetal imaging data, we used a parametric model of the resting‐state fMRI (rs‐fMRI) BOLD signal and neural event generation process to test and validate our method against a known ground truth. Finally, we examined 11 widely used rs‐fMRI denoising strategies on real clinical fetal fMRI data and compared their efficacy in mitigating motion effects.

## 2 MATERIALS AND METHODS

### 2.1 Participants and image acquisition

Data for this study were collected from in utero MRI scans acquired during routine clinical examination at Vienna General Hospital (AKH). The study protocol was approved by the institutional ethical boards at AKH and the Medical University of Vienna, and the research was conducted according to the principles expressed in the Declaration of Helsinki. All pregnant women were scanned without contrast agents, sedation, or breath‐hold. Subject exclusion criteria were: (a) known or diagnosed any neurological pathologies in the fetus, (b) multiple‐gestation pregnancy. For the final analysis, we used data from 70 unique fetuses with post‐menstrual gestational age (GA) ranging from 19 weeks 5 days to 39 weeks 2 days with an average of 28 ± 0.6 weeks. For each fetus, structural and functional MR images were acquired using a 1.5 T clinical scanner (Philips Medical Systems, Best, Netherlands) with a sensitivity encoding (SENSE) cardiac coil with five elements and the parameters described below.

(a) Structural imaging protocol: multiple consecutive T2‐weighted scans were acquired in approximate axial, coronal, and sagittal planes of the fetal brain with: TR=3s, TE = 80–140 ms, 0.65–1.7 mm in‐plane resolution, 3–5 mm slice thickness and acquisition time between 13.46 and 41.19 s. These scans were used to obtain individualized segmentations as will be explained in the next section.

(b) Functional imaging protocol: BOLD images were acquired with the following parameters: TR/TE = 3000/50 ms, matrix size of 144 × 144, in‐plane resolution = 1.74 × 1.74 mm^2^, slice thickness = 3 mm, flip angle = 90°, and 96 volumes per acquisition. During each TR interval, 18 slices were acquired with interleaved slice ordering (1–4–7…2–5–8…3–6–9…) to minimize cross‐talk between adjacent slices.

The total imaging time per subject per session was 45–60 min with an expert fetal neuroradiologist present to check for sufficient image quality and artifacts. fMRI acquisition time was around 7 min.

### 2.2 Data preprocessing

Structural data were first preprocessed with an in‐house built pipeline, as previously described (Schwartz et al., 2021). This pipeline is composed of the following steps: (1) denoising (Coupé et al., 2008) and in‐plane super‐resolution (Dong et al., 2016), (2) brain masking (Ebner et al., 2018), (3) N4 bias field correction with intensity normalization (Tourbier et al., 2017; Tustison et al., 2010), (4) motion correction and 3D reconstruction with isotropic super‐resolution (Ebner et al., 2018), and (5) rigid alignment of the resulting volumes to a common reference space (Gholipour et al., 2017). Segmentation of the isotropic volumes was performed by nonrigid mapping of a publicly available spatiotemporal atlas of fetal brain anatomy (Gholipour et al., 2017) to the individual cases. To account for variability in development, as well as limits in the accuracy of estimating the exact date of conception, atlases that spanned the prior and consecutive weeks of the estimated GA of each case were also used and merged using a label fusion technique (Wang et al., 2012).

Functional data were processed using ANTs (Tustison et al., 2010), NiftyReg (Ebner et al., 2018), AFNI (Cox, 1996) and in‐house scripts for preprocessing the rs BOLD data. Common elements of preprocessing included: (1) N4 bias field correction, (2) motion correction using an iterative method optimized for fetuses to correct for large and small head movements (Taymourtash et al., 2022), (3) removal of the spikes that occur sporadically in space and time, and (4) intensity normalization. After the application of each specific nuisance regression pipeline with and without censoring, additional common preprocessing steps including temporal high‐pass filtering with a cutoff frequency of 0.008 Hz was applied to the demeaned and detrended BOLD time series. We did not apply slice timing correction during preprocessing, as it has recently been suggested that temporal interpolation of voxel signals prior to realignment may artificially reduce motion estimates (Power et al., 2017). Individual FC analysis was performed in the native functional space and subject‐specific anatomical masks were mapped to this space using a rigid transformation computed between each individual T2w super‐resolution volume and the first frame of the realigned fMRI sequence.

Nuisance regression strategies were evaluated in this study variably incorporated six realignment estimates derived from motion correction (accounting for translations and rotations in three directions), their temporal derivatives and quadratic expansion, tissue‐specific signals extracted from the subject‐specific anatomical masks, global signal regression (GSR), aCompCor, and tCompCor (Behzadi et al., 2007). Censoring approaches were based on the voxels' signal intensity and the framewise displacement (FD). The former was performed using AFNI's 3dToutcount which flags any voxels in a TR that have signal intensity greater than 3 standard deviations from the other voxels in the brain mask. If more than 5% of the voxels in a volume are called outliers, that volume is marked as contaminated. For the latter, as in Satterthwaite et al. (2013), we calculated FD using the root mean squared volume‐to‐volume displacement of all brain voxels measured from the six head motion parameters. It should be noted since the fetal brain grows significantly during gestation, the radius of the brain cannot be assumed fixed for the whole population and unlike the adult studies, it needs to be estimated individually. Therefore, the rotational displacements were converted from degrees to millimeters by fitting a sphere to data using the least square method and calculating displacement on the surface of the estimated sphere. Table S1 outlines different strategies analyzed here. The code used for the analysis and experiments in this paper is publicly available at: https://github.com/cirmuw/fetalfMRIproc.

### 2.3 Quantifying residual motion artifacts in fetal FC fMRI

**FIGURE 1:**
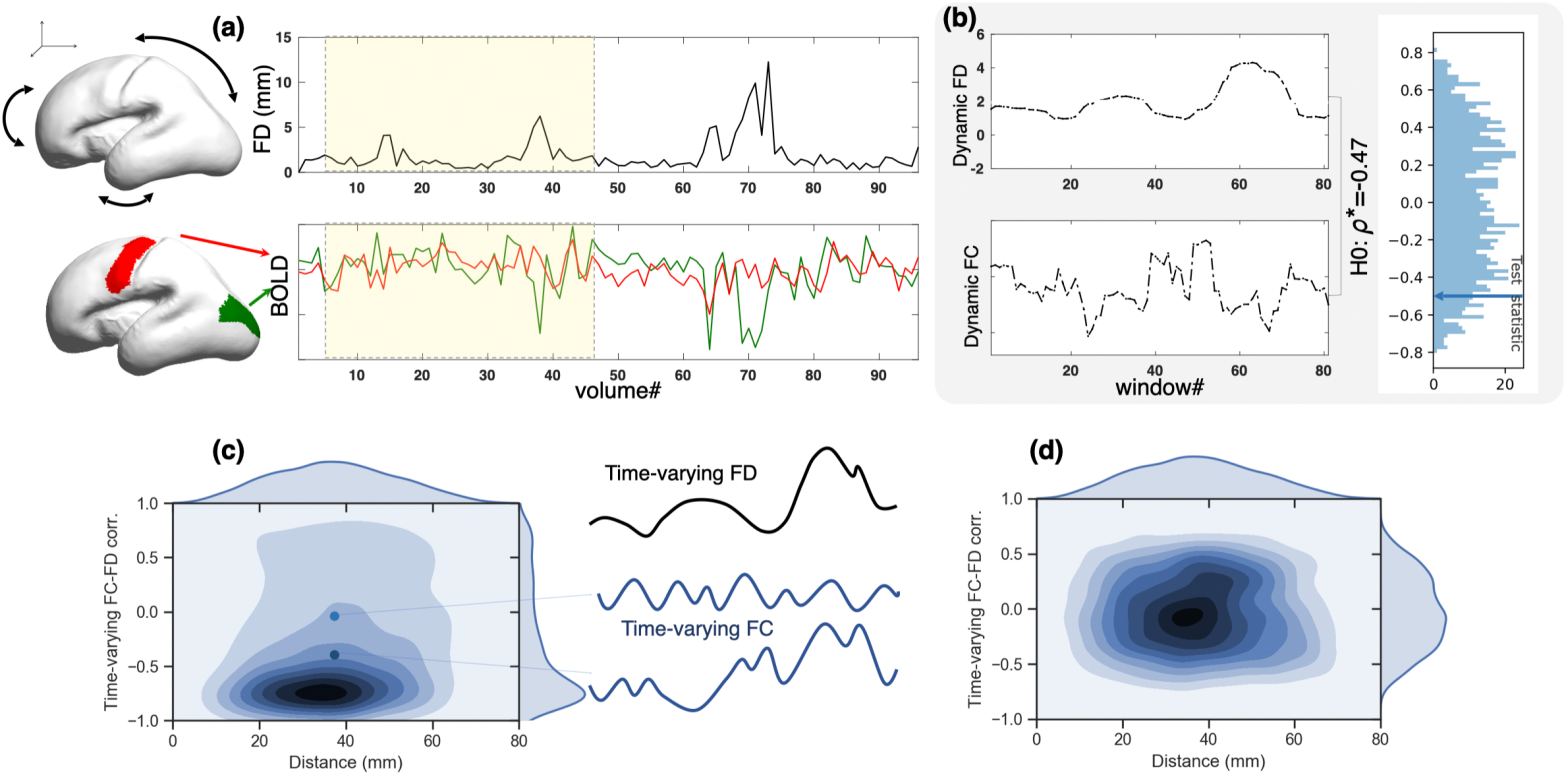
Illustration of the systematic approach to measure the relationship between functional connectivity (FC) and motion at the subject level. The average time series of BOLD signals from cortical ROIs are extracted. Time‐varying FC and framewise displacement (FD) are computed over sliding windows and the correlation coefficient is measured between them (a). To evaluate the statistical significance of the time‐varying FC–FD relation, an appropriate null distribution is formed by generating surrogate FD time series and repeating the entire procedure (b). Comparing the true value of the time‐varying FC–FD relation to the null distribution determines the extent to which motion drives the changes in FC. Subject‐level density plot indicating the time‐varying FC–FD correlation. The two dots show two pairs of regions, the distance between these regions are the same for both pairs but one of them is highly affected by motion (bottom) as the correlation is around −0.5 and the other one (top) is not affected much since the correlation is around zero (c). An effective preprocessing approach shifts the distribution of time‐varying FC–FD correlations for all ROI pairs at around zero regardless of the distance between them (d).

The present objective is to assess and quantify the impact of irregular and potentially large fetal head motion on the quality of BOLD fMRI data at the subject level and evaluate alternative regression strategies for mitigating this impact. Our approach is because non‐stationary sources of motion, like fetus movement, can potentially induce changes in FC over time, and tests the null hypothesis that these changes are related to the quality/motion‐content of the data. In other words, we test whether the relationship between the observed changes in FC estimates and the measured FD significantly deviates from those that might have been obtained from motion time series that lack the precise original ordering. The steps of the proposed procedure are illustrated in Figure 1.

Given fMRI data for a set of regions, we first calculated FC by evaluating signal correlation for all region pairs, using only the signal within a time window of 138 s (=46 TR). By sliding the time window through the entire available signal time series, we obtain a dynamic sequence of correlation values for each connection (FC). The same sliding window is used to average the FD of the head. The correlation coefficient between the two resulting time series of time‐varying FC and FD captures the association of FC and head motion during the length of the fMRI acquisition.

Since spurious correlation is possible, we compare the measured correlation with an appropriate null model. We generate a null model of FC–FD correlations by using the inverse Fourier transform of the amplitude spectrum (the absolute value of the discrete Fourier transform) of the real FC and FD pairs, combined with generated uniformly distributed phase spectra. The resulting signals share statistical properties with the measured motion, but randomize the timing of fluctuations. Thus, the resulting FC–FD correlation values are due to random relationships, and can serve as a null model of the association between head displacement and FC. If the observed correlation exceeds the critical value of the null distribution corresponding to the chosen significance level, then it is considered as statistically significant. This suggests a meaningful and nonrandom relationship between fetal head motion (FD) and FC in the subject data, with a higher absolute correlation value indicating a stronger link between motion and FC alterations, while a correlation close to zero indicates a lack of significant association. Thus, to evaluate the impact of subject‐specific motion on individualized FC, we quantify the number of functional connectivities that exhibit a significant relationship with motion for each subject. A successful denoising method is expected to reduce this number.

### 2.4 A model for simulating fMRI BOLD signals

To evaluate methods that quantify the impact of motion on FC in in utero rs‐fMRI, we simulate data. This enables controlling the characteristics of BOLD signals and the concomitant noise. Following prior simulation models (Bush & Cisler, 2013, 2014), we utilized a comprehensive parametric computational model of rs‐fMRI following assumptions about the generation and the nature of the observed BOLD signal. The steps of the simulation are depicted in Figure 3. First, latent neural events are encoded as a binary‐valued sequence generated via a first‐order hidden Markov model with an underlying maximum frequency of $\upsilon_{g}$ and 5% neural activity (firing compared to rest condition). The sequence of neural events is then convolved with a canonical hemodynamic response function (HRF) to form the true (i.e., theoretically ideal) BOLD signal. The HRF is modeled using a linear combination of two Gamma functions: one for modeling the initial positive response and the other one for the later undershoot in the HRF (Friston et al., 2002). To transform the ideal BOLD signal into the acquired signal via the scanner, we apply a series of mappings as the observation process, accounting for neural activity pruning, physiological noise contamination, down‐sampling, thermal noise contamination, and normalization. We simulated a total of 96 observed time points with %5 neural activity, $\nu_{g}=20\mathrm{Hz}$, $\nu_{o}=1/3\mathrm{Hz}$; ${\mathrm{SNR}}_{\text{phys}}=6$, and ${\mathrm{SNR}}_{\text{scan}}=9$. Neural events were mapped onto a BOLD signal via the canonical HRF (well‐approximated by a double gamma distribution with time‐to‐pick 6s) and Cholesky factorization was used to generate varying degrees of correlated neural activity.

Now consider two simultaneously recorded zero mean time series $X=\left(X_{1},\ldots ,X_{N}\right)$ and $Y=\left(Y_{1},\ldots ,Y_{N}\right)$ with the same observation process, and let ${\mathrm{cc}}_{n}$ be the sliding window correlation coefficient between $X_{n}$ and $Y_{n}$:

(1) $${\mathrm{cc}}_{\mathrm{xy}}\left[n\right]=\frac{\frac{\mathrm{TR}}{w}\sum_{i=n-\Delta }^{n+\Delta }x_{i}y_{i}-{\bar{x}}_{n}{\bar{y}}_{n}}{\sqrt{\frac{\mathrm{TR}}{w}\sum_{i=n-\Delta }^{n+\Delta }x_{i}^{2}-{\bar{x}}_{n}^{2}}\sqrt{\frac{\mathrm{TR}}{w}\sum_{i=n-\Delta }^{n+\Delta }y_{i}^{2}-{\bar{y}}_{n}^{2}}}$$

where $w=\left(2\Delta +1\right)$ TR is the window length in seconds, i sums only over the timepoints inside the window, and ${\bar{x}}_{n}$, ${\bar{y}}_{n}$ are the local averages inside the window at position n. Note that ${\mathrm{cc}}_{n}$ is allowed to depend on time n, that is, to be dynamic. This calculation is then repeated for all values of n, yielding the correlation time series $\mathrm{cc}={\mathrm{cc}}_{1},\ldots ,{\mathrm{cc}}_{L}$, where L is the largest integer such that L≤N−2Δ−1. A straightforward estimate of FD is obtained by down‐sampling the added physiological noise in the observation process with the rate of $d=\upsilon_{g}/\upsilon_{o}$, where $\upsilon_{o}$ is the frequency of observation ($\mathrm{TR}=1/\upsilon_{o}$). Subsequently, time‐varying FD $\mathrm{fd}={\mathrm{fd}}_{1},\ldots ,{\mathrm{fd}}_{L}$ is formed by calculating the local average in successive windowed segments of FD. In case of true correlation due to synchronized neural activity, the matrix of two generated ideal BOLD signals is first multiplied with the upper triangular matrix obtained from the Cholesky decomposition of the desired correlation matrix R, and then it goes through the observation process.

Having specified a model for the time‐varying FC (${\mathrm{cc}}_{n}$) and time‐varying FD (${\mathrm{fd}}_{n}$), we can formulate the problem of quantifying the impact of motion on FC in formal terms. Specifically, the absence of a relationship corresponds to the null hypothesis $H_{0}$: $\rho^{*}=0$, and its presence corresponds to the alternative hypothesis $H_{1}$: $\rho^{*}\neq 0$. The test statistics will be:

(2) $$t=\frac{r\sqrt{N-\left(2\Delta +1\right)-2}}{\sqrt{1-r^{2}}}$$

where r is the observed correlation between the ${\mathrm{cc}}_{n}$ and ${\mathrm{fd}}_{n}$, and t follows Student's *t* distribution with $N-\left(2\Delta +1\right)-2$ degrees of freedom.

## 3 RESULTS

In this section, we first evaluate the performance of the widely used QC–FC benchmarking method in a fetal cohort and examine its strengths and weaknesses in the context of in utero fMRI. We then evaluate if a novel approach addresses the limitations identified during the benchmarking evaluation and validate it using simulated data with known ground truth (i.e., the ideal BOLD signal with controlled levels of noise and neural activity). Finally, we evaluate the proposed method on real fetal fMRI and compare the efficacy of different existing regression strategies in mitigating the residual effects of motion at both subject‐ and group levels.

### 3.1 Evaluation of the QC–FC in fetal rs‐fMRI

**FIGURE 2:**
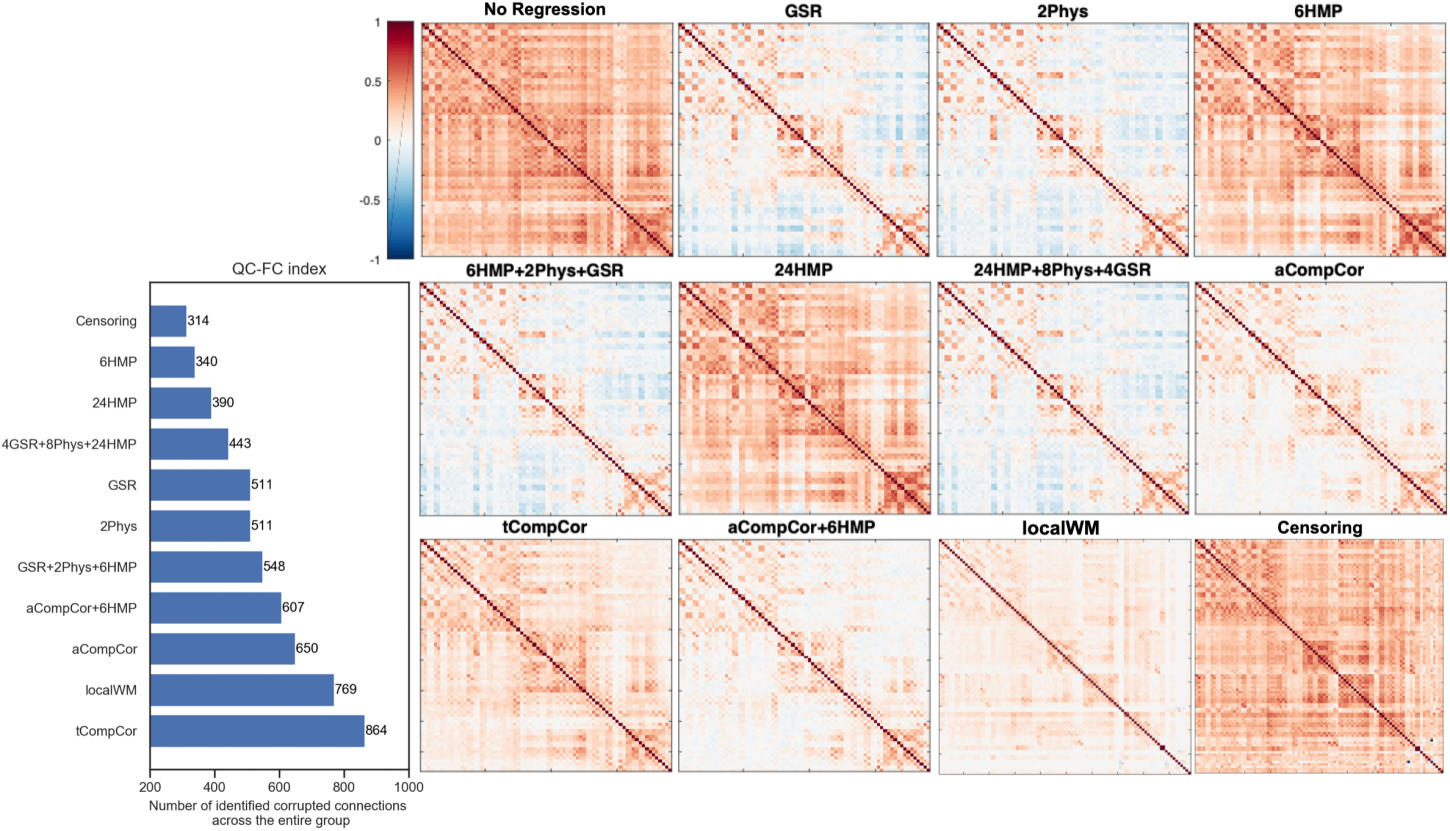
Evaluation of the widely used quality control measure (QC–FC index) in our fetal cohort. Panel (a) presents the number of detected edges significantly related to the motion in the entire cohort and panel (b) shows the corresponding group‐wise average functional connectivity maps. According to this measure, fewer significant edges indicate better performance of a regression strategy in mitigating motion effects, and thus the 6HMP model is the most successful method, however, its resulting FC map is very noisy.

The QC–FC correlation is a widely used metric to assess the relationship between motion and FC on a group level. For each pair of regions, it calculates the correlation between the average motion (meanFD) across subjects and the FC value of that edge. If this correlation is high and statistically significant, the edge is identified as motion corrupted for the entire group. Figure 2 presents the number of edges out of the total of 4753 edges connecting 98 ROIs derived from a spatiotemporal fetal atlas (Gholipour et al., 2017) with significant QC–FC correlations (p<.05, uncorrected) along with the resulting FC maps after applying each of the 12 regression strategies (see supplementary material for the distribution of QC–FC correlations). The bar plot suggests that regression models relying on the motion parameters (i.e., 6HMP and 24HMP) better minimize the effect of motion on FC compared to the other models across the entire group. Specifically, only 340 (7%) and 390 (8%) connections were found to be significantly associated with motion for 6HMP and 24HMP models respectively. Nevertheless, their corresponding FC maps show remaining influence of motion and deterioration of data quality following the application of these methods. On the other hand, although the application of, for example, aCompCor appears to improve FC maps for the entire group, it ranks among the poorest models based on the QC–FC index. These observations suggest a potential mismatch between the effectiveness of regression models as measured by the QC–FC index and the actual quality of FC maps obtained after applying these models.

### 3.2 Realistic rs simulation

Having recognized the limitations of the QC–FC metric in fetal data and the potential for false reassurance of data quality despite the presence of motion artifacts, we first used the parametric model for fMRI BOLD signal and neural event generation (Section 2.4) to validate the novel QC measure approach against a known ground truth. We generated simulated BOLD signals with varying degrees of correlated neural activity and noise contamination while the magnitudes of physiological and scanner noise were determined by the ratios calculated in Krüger and Glover (2001).

**FIGURE 3:**
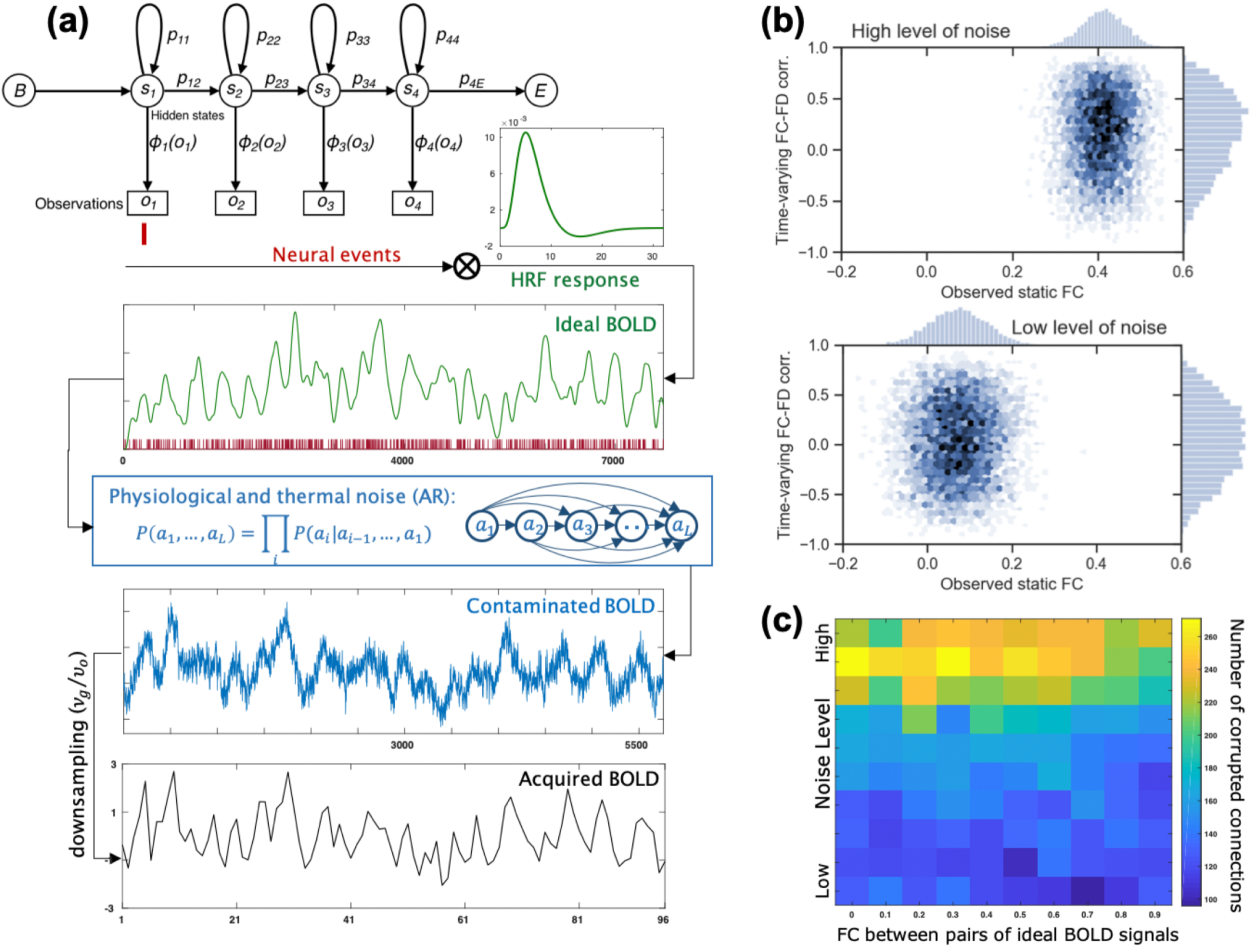
The process of simulating corrupted fMRI. Panel (a) depicts the generation from neural events, to the corresponding un‐corrupted BOLD signal, a noise contaminated BOLD signal, and finally a BOLD signal as acquired in fMRI. Panel (b) depicts the result of our quality control measure for two simulated data sets with low and high levels of contamination but the same correlation between the true BOLD signals. Note that when the level of contamination increases, more connections are identified as corrupted and the observed correlations inflate (type‐1 effect). Panel (c) shows the number of identified contaminated connections for different levels of noise and different values of the true BOLD signal functional connectivity.

Figure 3b illustrates the resulting distribution of time‐varying FC–FD correlation values for two datasets with high‐ and low contamination levels generated by the (${\mathrm{SNR}}_{\text{phys}}$, ${\mathrm{SNR}}_{\text{scan}}$) magnitudes of (2, 3) and (12, 18), respectively. Each dataset consists of 5000 pairs of BOLD signals with no correlation between their neural activity. As expected, with increasing noise, more connections were identified as corrupted (205 and 825, respectively). This is evident from the shift in the distribution of resulting values, which moves from being centered at 0.02 to around 0.318, as shown on the y‐axis. Despite the true correlations between the simulated pairs of signals being set to zero, increasing the level of contamination inflated the observed connectivity values from around 0.07 to 0.41, as shown on the x‐axis (known as the type‐1 motion effects). Figure 3c depicts the number of detected corrupted connections at different levels of contamination and different levels of actual neural event synchronicity. Consistent with our hypothesis, our method detects corrupted connections with increasing noise levels, but does not erroneously classify actual synchronization as motion artifact.

### 3.3 Real in utero fMRI: Single‐subject assessment

**FIGURE 4:**
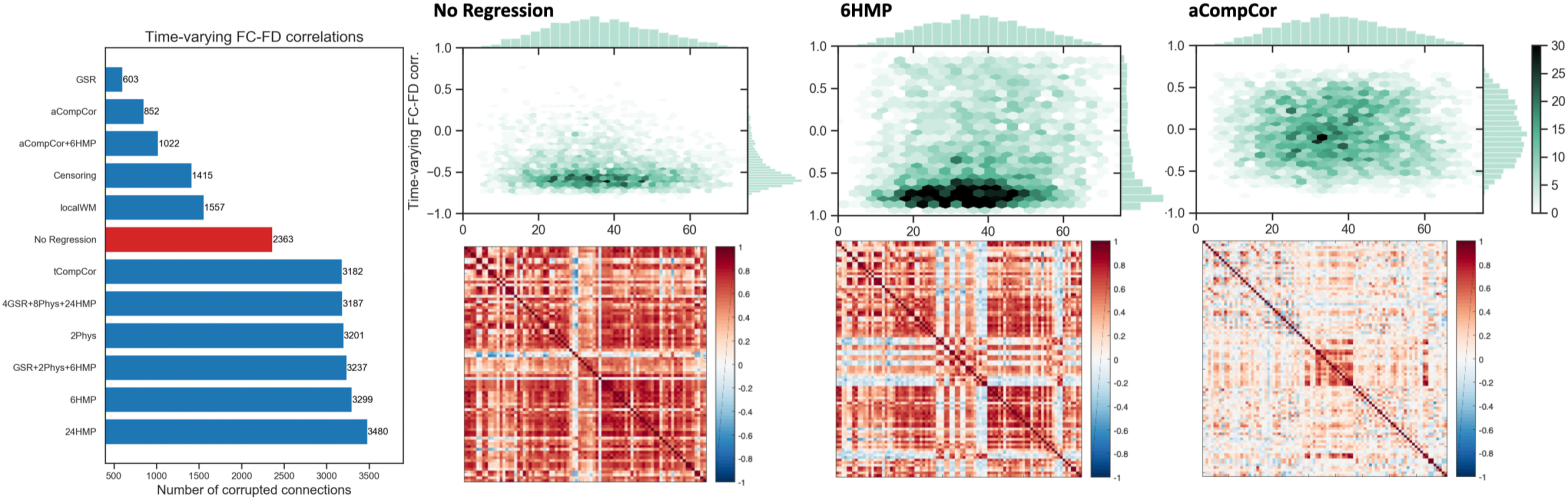
Using time‐varying FC–FD relation to evaluate the quality of data at the subject level. Right panel demonstrates the resulting measure, with the y‐axis representing time‐varying FC–FD correlations and the x‐axis showing the subject‐specific Euclidean distance separating each pair of the parcels. A successful model should yield a higher density of data points around the zero axis (indicating fewer signification correlations), regardless of the distance between the parcels. These correlations are compared to the null distributions, and the best performing models for this specific subject are GSR and aCompCor (left panel).

To quantify the impact of residual motion on each connection of a single subject, we introduced a statistical method to assess if the changes in connectivity strength are modulated by the motion (Section 2.3). Results obtained with this method for a typical fetus that exhibited continuous head movements during the acquisition are shown in Figure 4 and Supplementary Figure S2. Although the motion‐induced variance in the BOLD signal is not directly observable in the FC map (lower plots), we can clearly discern spuriously inflated correlations that are correlated with motion in the time‐varying FC–FD plots (upper plots).

Without conducting nuisance regression, %49.7 of connections (with median absolute time‐varying FC–FD correlations of 0.533) were significantly related to the observed motion. Anatomical PCA‐based correction models (localWM, aCompCor+6HMP, and aCompCor) improved the quality of data and considerably decreased the number of motion‐corrupted connections to %33, %21, and %18 with the median absolute time‐varying FC–FD correlations of 0.35, 0.28, and 0.26, respectively. GSR provided further improvement yielding only %13 significantly corrupted connections (median absolute correlations of 0.21). In contrast, regression models relying on the motion parameters alone (6HMP and 24HMP), tissue‐averaged physiological regressors, and tCompCor aggravated the effects of motion on FC as the percentage of corrupted connections was increased to %67−%73 following the application of these models.

**FIGURE 5:**
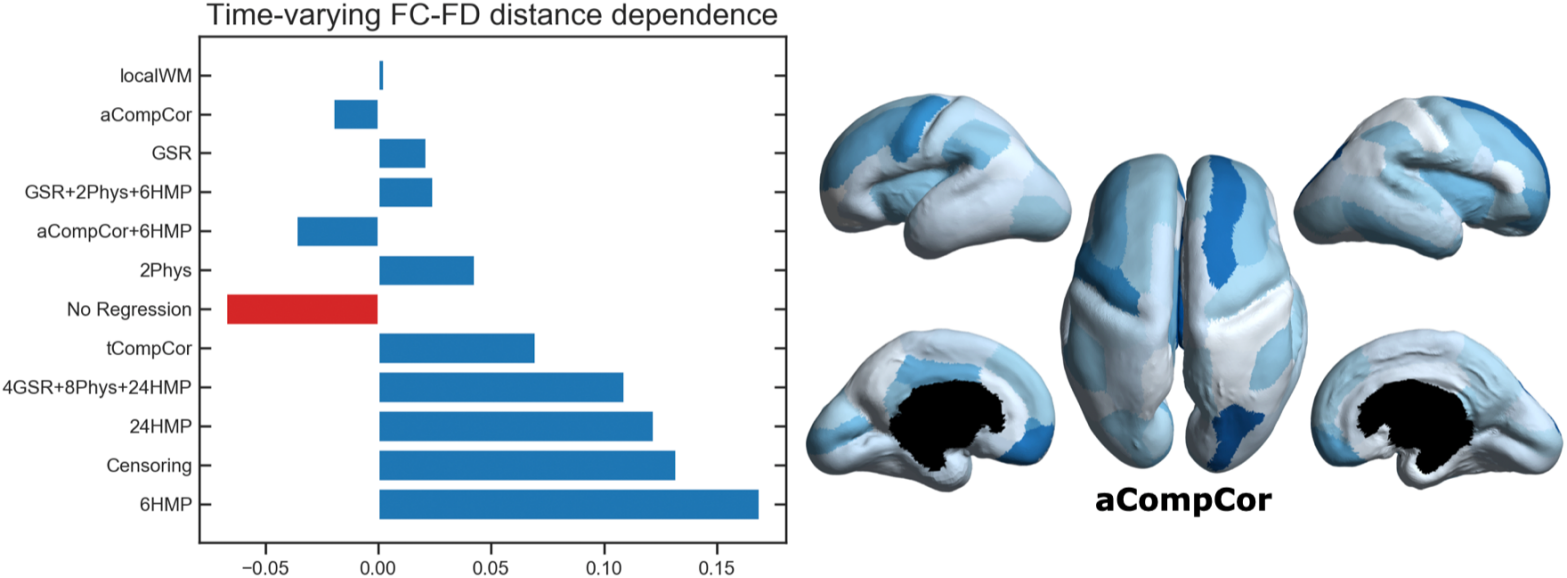
Left: Assessment of distance dependence in time‐varying FC–FD correlations using various pipelines on a representative fetus at a GA of 29w + 3d. Spearman rank correlation was computed between time‐varying FC–FD correlations and the Euclidean distance separating the associated regions. Right: Uncertainty map of brain regions for the same fetus after applying aCompCor model. The degree of uncertainty is quantified as the number of edges significantly influenced by motion.

The residual relationship between time‐varying FC–FD correlations and inter‐regional distance at the subject level is shown in Figure 5. A successful denoising model should remove the association between motion and the FC connecting a pair of regions regardless of the distance between them. As such, we calculated the Euclidean distance between the centers of ROI pairs and measured the Spearman's rank correlation coefficient between time‐varying FC–FD correlations and their corresponding distances. Again, anatomical PCA‐based correction methods (localWM, aCompCor) exhibited lower residual values, reducing the distance dependence observed in the data from −0.0675 to 0.0025 and −0.0200, respectively. See supplementary materials for results using *p* < .05 FDR‐corrected (Figure S3), as well as the distributions of time‐varying FC–FD correlations for alternative regression models (Figure S4).

### 3.4 Real in utero fMRI: Group‐level assessment

**FIGURE 6:**
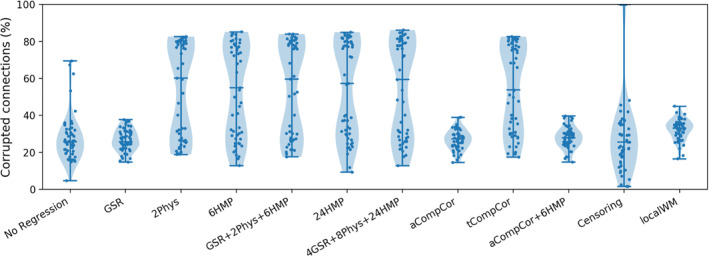
Comparison of different regression models in mitigating the effects of motion artifacts on functional connectivity based on our proposed quality control measure. Performance is measured as the number of identified corrupted connections at the subject level. For the entire cohort, we calculated the proportion of remaining corrupted connections after applying each denoising model.

Figure 6 shows the distribution of connections detected to be corrupted (p<.05, uncorrected) across the entire group of 70 fetuses. Each data point represents a single fetus, with the evaluation performed after applying each of the 11 regression models listed in Table S1. Compared to QC–FC, the novel quality measure corresponds more closely to the quality of FC matrices in Figure 2b.

Similar to single subject evaluation, both aCompCor (%26.86, IQR = %6.76) and GSR (%26.62, IQR=%8.81) performed well across all subjects while the 4GSR + 8Phys + 24HMP model fared the worst (%60.10, IQR=%53.18). As expected, the regression models involving GSR introduced negative correlations to the FC maps (see Figure 2). The censoring model involved a costly trade‐off, where each censored volume enhanced data quality but came at the cost of shorter time series and variably reduced degrees of freedom across subjects. As a consequence, four subjects in our group had all their connections identified as corrupted due to the short time series remaining after censoring. Excluding these subjects improved the average of time‐varying FC–FD correlations across subjects from %29.67 (IQR=%18.87) to %23.93 (IQR=%20.06).

Finally, we compared the residual distance dependence between regression models for the entire group. Due to significant changes in brain size throughout gestation (see Figure S1), we computed inter‐regional distances on an individual basis. These distance vectors, alongside the time‐varying FC–FD correlations, were then pooled across the group for calculating the Spearman's rank correlation. The results revealed that, with the exception of the censoring model, which amplified the residual association from −0.0177 to −0.0366, all other models led to a significant decrease in this association (p<.001).

## 4 DISCUSSION

In this study, we presented a computational QC method that addresses the dynamics of FC and the non‐stationarity of fetal movement to detect corrupted FC at the individual subject level. Experimental evaluation on simulated‐ and real fetal fMRI data demonstrated that it outperforms state of the art QC approaches designed for adult data. Finally, we used the method to compare 11 widely used regression models for motion artifact removal in real fetal fMRI data, concluding that methods such as aCompCor or GSR do reduce motion contamination in FC better than approaches that take measured motion parameters into account.

A parametric model for the generation of corrupted fMRI BOLD signal from known neural event time series with varying levels of noise and neural activity enabled us to validate the proposed method with a known ground truth. Here, results show the accuracy and reliability of our method in identifying and flagging corrupted connections.

One critical consideration in fMRI studies of living human fetuses is the potential interplay between two factors: high levels of motion contamination and the ceiling effect (Power et al., 2015). The ceiling effect occurs when motion‐related increases in FC reach a maximum limit and saturate the connectivity maps for nearly the entire cohort, as observed in fetal data. Consequently, when computing the QC–FC metric, the correlation with the widely varying average motion across subjects often fails to reach significance due to the limited variability in saturated FC across subjects. Thus, the commonly used QC–FC correlations tend to approach zero, and only a limited number of connections are identified as motion corrupted (Figure 2a and Figure S1). This can potentially create a misleading reassurance of data quality despite the presence of motion artifacts and thus highlights the necessity of developing alternative measures to ensure reliable and accurate quality assessment in fetal samples. The QC–FC metric inherently assumes a uniform influence of motion on edge‐wise FC estimates across subjects (Kassinopoulos & Mitsis, 2022) and it is not practical to use it for single‐subject or small group analyses, as its reliability scales with the number of subjects involved (Ciric et al., 2017; Parkes et al., 2018; Power et al., 2014). As a result, QC–FC and similar approaches employing different outcomes such as modularity (Ciric et al., 2017) and dispersion index (Lydon‐Staley et al., 2019), face limitations when applied to in utero fMRI. The added variability in fetal fMRI due to substantial developmental changes further limits the effective use of QC–FC metric as a reliable group‐level index of data quality.

Assessing the relationship between FC (time‐varying FC) and fetal motion (time‐varying FD) using sliding windows is critical because FC may vary because of measurement noise, or because of non‐stationary neural activity such as dynamic brain states (Hindriks et al., 2016). Therefore, a statistical test against an appropriate null model of the link between FC and FD is required to detect motion corruption.

We demonstrated the mismatch between the QC–FC outcome and the quality of fetal FC maps, and its adverse impact on the comparison of motion regression approaches. When comparing these models using the QC–FC metric, the resulting ranking tends to favor models that exhibit clear signs of artifacts in their resulting FC maps. Notably, regression of 6HMP is identified as the best‐performing strategy, while aCompCor is among the worst. This discrepancy underscores the limitations of the QC–FC metric when applied to fetal data. In contrast, the ranking of regression models in fetal data using our proposed method aligns with previous studies (Ciric et al., 2017; Graff et al., 2022; Parkes et al., 2018; Pruim et al., 2015; Satterthwaite et al., 2013; Yan et al., 2013) showing relatively poor performance of simple regression of motion parameters even when adding expansion terms. Censoring generally performed well across subjects, while it results in the exclusion of more individual fetuses due to insufficient length of the time series left after censoring. Both anatomical PCA‐based correction models (aCompCor and localWM) effectively mitigated residual motion and distance‐dependence effect across the entire group, and notably PCA‐based regressors, unlike in GSR, do not necessarily mandate negative correlations in FC maps (Carbonell et al., 2011). While our study focuses only on fetal rs‐fMRI, we cannot generalize the results of our metric to adult rs‐fMRI without further investigation.

This article has several limitations. It is not a comprehensive evaluation of all denoising strategies in use, but of those commonly used at present. We did not analyze ICA‐based regression models since those methods have not been tailored for the fetal population so far. The available algorithms for automatic identification of noise‐related components such as ICA‐AROMA (Pruim et al., 2015) were trained on adult fMRI and rely on registering the spatial map of ICs to an adult brain template like MNI152 (Fonov et al., 2011) which significantly differs from spatiotemporal atlases of fetal brain that are specific for each week of gestation. Our analysis focused on a single dataset of 70 in utero fMRI and we only considered a single fetal brain parcellation (Gholipour et al., 2017), primarily due to the scarcity of open‐access fetal data available. Although the exhibited motion characteristics and the performance of different regression models can depend on the data, the developed method is applicable to diverse datasets across different parcellation resolutions. The age‐related variability in fetal fMRI data (Ji et al., 2023), coupled with the developmental changes in brain structure and function, may introduce additional challenges for QC measures and motion correction strategies. As such, future research endeavors should aim to explore the impact of fetal age on the efficacy of preprocessing techniques and the interpretation of FC findings. Additionally, in this article, we did not make specific recommendations for a particular regression model or the censoring threshold to account for the effects of motion on in utero FC. Importantly, as noted by Power and colleagues (Power et al., 2014), all datasets should be evaluated independently for proper denoising strategy and FD thresholds. While beyond the scope of the current study, a systematic evaluation across multiple datasets is needed in future works.

## 5 CONCLUSIONS

The significant impact of both fetal head motion and preprocessing choices on FC estimates emphasizes the importance of QC methods tailored to the unique characteristics of fetal fMRI data. We propose a method extending QC–FC to the individual subject level in fetal scans exhibiting substantial motion. As it becomes common to use hundreds of scans for a single fetal study, manually assessing the quality of data is no longer feasible. Our work provides investigators with a means to evaluate and compare available de‐noising approaches, on both real and simulated data.

## CONFLICT OF INTEREST STATEMENT

Georg Langs is co‐founder and shareholder of contextflow GmbH and research grant holder from Novartis, NVIDIA—Both are unrelated to the topic of this article.

## Supporting information


**DATA S1:** Supporting Information.

## Data Availability

Due to the sensitive nature of the clinical MRI/fMRI data utilized in this research, strict confidentiality measures have been implemented to protect the privacy and anonymity of the study participants. The raw data generated from the clinical imaging procedures will not be shared or made publicly accessible. This decision is in accordance with ethical guidelines and ensures the security of sensitive medical information. Requests for access to the processed data may be considered under specific circumstances, subject to approval by the relevant institutional review board and in compliance with applicable legal and ethical standards. For inquiries regarding data access, please contact georg.langs@meduniwien.ac.at. The code for preprocessing, simulation, and quality control of fetal fMRI is publicly available at https://www.github.com/cirmuw/fetalfMRIproc.

## APPENDIX A SPECTRAL DECOMPOSITION OF THE CORRELATION COEFFICIENT

### A.1

The correlation coefficient can be decomposed into frequency components by Fourier transformation of the voxel time courses. Let $f\left(t\right)$ and $g\left(t\right)$ represent the signal intensity of two voxels as a function of time, defined over the interval 0,N. Furthermore, the mean signal of each function has been subtracted. Using the inverse Fourier transform, we can write:

(A1)
$$\begin{array}{ll} & f\left(t\right)=\sum_{n=1}^{N-1}\omega_{n}e^{i2\mathrm{\pi nt}/N} \end{array}$$

(A2)
$$\;g\left(t\right)=\sum_{n^{'}=1}^{N-1}\lambda_{n^{'}}e^{i2\pi n^{'}t/N}$$

where N−1 refers to the number of frequency components and $\omega_{n}$, $\lambda_{n}$ refer to the complex‐valued frequencies of the Fourier transform of $f\left(t\right)$ and $g\left(t\right)$, respectively. Note that $\omega_{0}=\lambda_{0}=0$ in our case. The correlation coefficient, cc, involving $f\left(t\right)$ and $g\left(t\right)$ is defined as:

(A3)
$$\mathrm{cc}=\frac{\sum_{t=0}^{N-1}f\left(t\right)g\left(t\right)}{D}$$

where the denominator, D, is given by:

(A4)
$$D=\sqrt{\sum_{t=0}^{N-1}f^{2}\left(t\right)\sum_{t=0}^{N-1}g^{2}\left(t\right)}$$

Inserting Equations A1 and A2 into Equation A3 yields:

(A5)
$$\mathrm{cc}=\frac{\sum_{t=0}^{N-1}\sum_{n=1}^{N-1}\omega_{n}e^{i2\mathrm{\pi nt}/N}\sum_{n^{\prime }=1}^{N-1}\lambda_{n^{\prime }}e^{i2\pi n^{\prime }t/N}}{D}$$

Rearranging the order of summation and combining terms gives:

(A6)
$$\mathrm{cc}=\frac{\sum_{n=1}^{N-1}\sum_{n^{\prime }=1}^{N-1}\omega_{n}\lambda_{n^{\prime }}\sum_{t=0}^{N-1}e^{\left[i2\pi \left(n+n^{\prime }\right)t\right]/N}}{D}$$

The sum over t simplifies owing to the orthogonality condition:

(A7)
$$\sum_{t=0}^{N-1}e^{\left[i2\pi \left(n+n^{\prime }\right)t\right]/N}=N\delta_{n+n^{\prime },\mathrm{mN}}$$

where $\delta_{i,j}$ is the Kronecker delta symbol and m can be any integer. Since in our case:

(A8)
$$0<n+n^{\prime }<2N$$

m=1 is the only possibility in Equation A7 for a nonvanishing contribution. Hence, we can write Equation A6 as:

(A9)
$$\mathrm{cc}=\frac{\sum_{n=1}^{N-1}\sum_{n^{\prime }=1}^{N-1}\omega_{n}\lambda_{n^{\prime }}N\delta_{n+n^{\prime },N}}{D}$$

which simplifies to:

(A10)
$$\mathrm{cc}=\frac{\sum_{n=1}^{N-1}\omega_{n}\lambda_{N-n}N}{D}$$

Since the original voxel‐time courses are real‐valued functions, the complex Fourier components satisfy the symmetry condition:

(A11)
Reλn=ReλN−n

(A12)
Imλn=−ImλN−n

The expression for the correlation coefficient in Equation A10 becomes:

(A13)
cc=N∑n=1N−1ReωnReλN−n−ImωnImλN−nD

and hence:

(A14)
cc=N∑n=1N−1ReωnReλn+ImωnImλnD

We are now able to define the spectral decomposition of the correlation coefficient by:

(A15)
ccn=NReωnReλn+ImωnImλnD

with:

(A16)
$$\mathrm{cc}=\sum_{n=1}^{N-1}{\mathrm{cc}}_{n}$$

In the following, we include superscripts to label the two voxels that are correlated. For an ROI, the average spectral decomposition of the correlation coefficient between a seed voxel, *s*, and all other voxels in the brain, *j*, can be defined by:

(A17)
$${\left\langle {\mathrm{cc}}_{n}^{\left(s,j\right)}\right\rangle }_{j}=\frac{1}{M}-\sum_{j=1}^{M}{\mathrm{cc}}_{n}^{\left(s,j\right)}$$

where the upper indexes *s* and *j* refer to the seed voxel and to all other voxels on the fcMRI map, respectively. The index *j* labels all voxels in the brain that have significant correlation; that is,

(A18)
$${\mathrm{cc}}^{\left(s,j\right)}>\mathrm{thr}$$

for all *j* voxels on the fcMRI map. The term thr represents a threshold at the appropriate significance level.
