# Supplementary material for: Measuring the effects of motion corruption in fetal fMRI
Source: Hum Brain Mapp. 2025 Jan 23;46(2):e26806. doi: 10.1002/hbm.26806 (PMC11755121; doi:10.1002/hbm.26806)
Supplement: Supplementary file 1 — DATA S1: Supporting Information. [file HBM-46-e26806-s001.docx]

## Supplementary Information

[[Image]]

**Figure S1: a) Estimating the radius of the brain for each individual fetus. c) The residual distance-dependence effect of motion artifact for the entire group after applying different regression models. As the fetal brain grows significantly during gestation, the radius of the brain and the Euclidean distance separating a pair of regions were estimated individually for every fetus, and normalized distances were used to measure the residual distance-dependence effect for the entire group.**

**Table S1: Ten nuisance regression strategies evaluated here.**

| **Strategy** | **Summary of regressors** | **#R** |
| --- | --- | --- |
| **1:** GSR | Mean time-series averaged across the entire brain | 1 |
| **2:** 2Phys | Two physiological time-series computed across white matter (WM) and cerebrospinal fluid (CSF). | 2 |
| **3:** 6HMP | 6 motion parameter estimates derived from realignment. | 6 |
| **4:** 6HMP + 2Phys + GSR | 6 motion parameter estimates, 2 physiological compartments and GSR | 9 |
| **5:** 24HMP | 6 motion parameters, their temporal derivatives, together with quadratic expansions of parameters and derivatives. | 24 |
| **6:** 24HMP + 8Phys + 4GSR | Quadratic expansion of model 4: 9 regressors, their derivatives, quadratic terms, and squares of derivatives. | 36 |
| **7:** aCompCor | 5 principal components each from the WM and CSF Behzadi et al. (2007) | 10 |
| **8:** tCompCor | 6 principal components from high-variance voxels Behzadietal.(2007) | 6 |
| **9:** localWM | voxel-varient principle component extracted from a local neighborhood of each voxel Cox (1996) | 1 |
| **10:** Censoring | The entire volumes marked as contaminated by thresholding a given participant’s FD Power et al. (2012) | $n$ |

**Notes. $n$ indicates a variable number of regressors across participants.**

[[Image]]

**Figure S2: A carpet plot (upper panel) depicting the BOLD signal of gray matter voxels over time for a single fetus. Each row of this plot represents the time course of a gray matter voxel. The lower panel depicts the estimated framewise displacement (FD), a measure of head motion, throughout the fMRI scan.**

[[Image]]

**Figure S3: Comparison of motion-corrupted connections in fMRI data without with multiple comparison corrections using false discovery rate (FDR; Benjamini and Hochberg, 1995).**

[[Image]]

**Figure S4: Evaluation of different regression models based on time-varying FC-FD correlations for a representative fetus.**

### Analysis on a separate cohort of fetuses:

We performed an additional experiment on a separate cohort of 21 fetuses with gestational ages ranging from $18^{w}+0^{d}$ to $39^{w}+1^{d}$ (mean: 28.67 weeks). The imaging protocols remained consistent with the main cohort in the paper, except for the number of volumes per series which increased to 140 in this new cohort. We conducted the same preprocessing procedures for both anatomical and functional images, and derived 39 cortical ROIs per hemisphere for each subject from an automatic atlas-based segmentation using a spatio-temporal atlas of fetal brain (Gholipour et al., 2017). The distribution of the estimated framewise displacement (mm) for each subject is depicted in Fig. S5. Fetal head movements quantified by either the mean or the maximum of the framewise displacement did not correlate with fetal age (p-value = 0.199 and 0.303 respectively).

[[Image]]

**Figure S5: The distribution of the estimated framewise displacement (mm) for each subject. Subjects were sorted based on their gestational age such that S01 is the youngest fetus in this new cohort with the age of 18w+0d and S21 is the oldest one with 39w+1d. There was no significant correlation between the age and either the average (p-value = 0.199) or the maximum FD (p-value = 0.303).**

Fig. S6 and Fig. S7 show the resulting distribution of tvFC-FD correlation values and the corresponding FC maps for two fetal subjects with relatively high (S3) and low (S12) motion contamination during fMRI acquisition. Subject S3, with a gestational age of 21w+5d, showed an average framewise displacement of 10.05mm (IQR = 6.38mm). Our subject-specific quality control metric indicates most of the connections ($2529/3003=84.22\%$) were significantly affected by motion in this subject, and none of the regression models could successfully mitigate this strong effect in the data. As observed in the barplot, the best performing model in mitigating the association between motion and FC could only decrease the number of affected connections to 77%. Consistent with the main results of the paper, the simple regression of motion parameters alone fared the worst (86%) and the resulting functional connectivities following the application of this model are likely to be heavily contaminated by motion. Censoring was not possible for this subject, as the remaining volumes after scrubbing was not enough to measure a statistically reliable FC.

[[Image]]

**Figure S6: Evaluation of data quality using the time-varying FC-FD correlation for the subject S03. This subject was moving a lot during acquisition with an average displacement of 10.05 millimeter (SD=7.68). Our subject-level metric shows non of the regression strategies were successful in removing the effect of motion corruption on functional connectivity as the number of identified corrupted connections were reduced from 2584 (86%) in preprocessed data (No regression) to only 2304 (77%) in the best strategy (4GSR+8Phys+24HMP).**

As another example, we present the resulting quantitative evaluation of alternative regression models using our proposed metric (tvFC-FD) for a typical fetus randomly selected from the middle quartiles of this new cohort (Fig. S7). This subject, S12, with a gestational age of 30w+0d, showed an average FD of 2.38mm (IQR=1.45mm) and 17 volumes detected as outliers based on the approach explained in the main manuscript (Section 2.2). As observed in Fig. S7, many connectivity values in the FC map without applying any regression model are spuriously inflated but it is not possible to identify which connection and to what extent. The proposed metric quantifies the quality of this FC map and indicates that for 2532 connections (84.3%), there is a statistically significant association between the FC and the exhibited motion by this subject so that their changes during acquisition correlates significantly.

Using the proposed metric, we can compare the efficacy of different regression models in mitigating such strong association for this subject. Here, the GSR model has reduced this association to 845 connections (28%) and aCompCor reduced it to 1096 connections (36.5%) as shown in Fig. S7. In terms of association with the distance, a successful model should correct the motion effects regardless of the distance between parcels, and it should not introduce a distance-dependence bias into the data. As observed in the figure, for this particular subject, aCompCor performed the best as the association between distance and correlation was not significant.

Note that we do not intend to make specific recommendations for a particular regression model to account for the effects of motion on in-utero functional connectivity. Instead, we present a quantitative approach to evaluate the degrees of motion corruption in data and compare available denoising models at the subject level.

[[Image]]

**Figure S7: Evaluation of data quality using the time-varying FC-FD correlation for the subject S12. The average framewise displacement for this subject across the entire acquisition was 2.3 millimeter (SD=2.6). Our subject-level quality control metric shows the efficacy of each regression strategy in mitigating the effect of motion corruption on functional connectivity. The best strategy has been (4GSR+8Phys+24HMP) which has reduced as the number of corrupted connections were reduced from 2532 (84%) to only 802 (26%).**
